# Trends in primary care antibiotic prescribing during the implementation of national stewardship policies: Türkiye, 2011–2019

**DOI:** 10.3389/fmicb.2026.1762561

**Published:** 2026-05-22

**Authors:** Hakkı Öztürk, Bircan Kayaaslan

**Affiliations:** 1 Afyonkarahisar Provincial Health Directorate, Afyonkarahisar, Türkiye; 2 Department of Infection Diseases and Clinical Microbiology, Ankara Yıldırım Beyazıt Üniversitesi, Ankara Bilkent Şehir Hastanesi, Ankara, Türkiye

**Keywords:** antibiotic stewardship, antimicrobial resistance, AWaRe classification, primary care, rational use of antibiotics, Türkiye

## Abstract

**Background:**

Antimicrobial resistance is driven by inappropriate antibiotic use, and Türkiye has long shown one of the highest outpatient consumption rates in the WHO European Region. In response, the Ministry of Health implemented stewardship measures from 2014 onward, including the National Action Plan for Rational Drug Use, banning over-the-counter antibiotic sales, integrating e-prescribing with mandatory diagnostic coding, educational initiatives, and audit-feedback systems. This study evaluated temporal trends in primary care antibiotic prescribing in Türkiye between 2011 and 2019 and assessed the influence of these interventions.

**Methods:**

We performed a nationwide descriptive analysis using aggregated Prescription Information System (RBS) data covering all family-physician prescriptions (2011–2019). Outcomes included: (i) proportion of prescriptions containing systemic antibiotics (ATC J01), (ii) consumption in DID, (iii) use by ATC-3 class and AWaRe category, (iv) antibiotic share of drug volume and cost, and (v) patterns by age, sex, and diagnosis. Trends were summarized using annual percentages and DID values. The two pre-specified primary outcomes were the annual antibiotic prescription rate and total systemic antibiotic consumption (DID, ATC J01); other indicators were treated as secondary. Because the RBS captures only e-prescribed antibiotics, over-the-counter sales (legal in Türkiye until 2016) are not included. Segmented (interrupted time-series) regression with Newey–West HAC standard errors was applied to test for changes in level and slope at 2016, when prescription-only dispensing was fully enforced and mandatory ICD-10 coding was consolidated within the e-prescribing system.

**Results:**

Among 1.17 billion prescriptions, the share containing an antibiotic declined from 34.9 to 23.9%, while consumption fell from 42.3 to 31.9 DID (−25%). Beta-lactams showed the largest reductions, though Watch-group agents still formed nearly half of use in 2019, below the WHO ≥60% Access target. Pediatric prescribing decreased most: use in children aged 0–6 years dropped from 60 to 44% of visits. Elderly patients had the lowest rates. Diagnostic coding shifted away from nonspecific upper-respiratory infections. Antibiotics’ share of outpatient drug costs fell from 10.7 to 4.7%, yielding an estimated 1.7 billion TL savings in 2018. Segmented regression confirmed an acceleration of the decline after 2016: the slope changed by −0.69 percentage points/year for the prescription rate (95% CI − 1.44 to +0.06; *p* = 0.07) and by −0.80 DID/year for total consumption (95% CI − 0.88 to −0.72; *p* < 0.001) versus the pre-2016 trajectory.

**Conclusion:**

Türkiye achieved major reductions in primary care antibiotic use within a decade, temporally concordant with multifaceted stewardship policies whose individual contributions cannot be isolated by this descriptive design. However, outpatient levels remain above many EU countries, and Watch-group use is still high. Further progress requires stronger guideline adherence, prioritization of Access antibiotics, and wider use of point-of-care diagnostics.

## Introduction

Antibiotics are essential for the treatment of bacterial infections; however, their inappropriate use has contributed substantially to the global rise of antimicrobial resistance (AMR), threatening the effectiveness of these life-saving drugs (Salam et al., 2023). The World Health Organization (2004) estimates that more than half of all medicines worldwide are prescribed, dispensed, or sold inappropriately, with antibiotics constituting a major component of this problem (Ofori-Asenso and Agyeman, 2016). Inappropriate antibiotic use is associated with treatment failure, avoidable adverse drug reactions, increased healthcare expenditures, and the emergence and dissemination of antibiotic-resistant bacteria (Salam et al., 2023; Butler et al., 2022; Dadgostar, 2019).

To address these challenges, the WHO introduced the concept of Rational Use of Medicines (RUM) in the 1980s, defining it as the provision of the right medicine, in the right dose and duration, for the right patient, at the lowest possible cost to individuals and society (World Health Organization, 1987; World Health Organization, 2025). Antimicrobial stewardship programs operationalize these principles specifically for antibiotics, aiming to ensure that antimicrobials are prescribed only when clinically indicated and that the selected agent, dose, route, and duration are optimal (World Health Organization, 2019; Majumder et al., 2020). Such programs are widely recognized as a cornerstone strategy for slowing the spread of antimicrobial resistance and preserving the effectiveness of existing antimicrobial agents.

The public health urgency of antimicrobial resistance is further underscored by increasing reports of multidrug-resistant organisms across a wide range of infectious syndromes, including community-acquired respiratory tract infections, urinary tract infections, and healthcare-associated infections (Salam et al., 2023; Butler et al., 2022; Dadgostar, 2019). Although resistance patterns vary by pathogen, clinical setting, and geography, excessive and inappropriate antibiotic use in the community is a key driver of resistance selection pressure. Consequently, reducing unnecessary outpatient antibiotic exposure is considered a critical upstream intervention to limit the emergence and spread of multidrug-resistant bacteria.

Türkiye has historically faced a particularly pressing challenge in this regard. In the early 2010s, the country was reported to have one of the highest outpatient antibiotic consumption rates in the WHO European Region (Aksoy et al., 2021). Multinational analyses demonstrated that Türkiye’s overall antibiotic use exceeded that of many European countries, with a strong preference for broad-spectrum agents. Notably, amoxicillin-clavulanate alone accounted for approximately 30% of outpatient antibiotic consumption, a proportion substantially higher than that observed in most comparator countries (Westerling et al., 2020). This high level of antibiotic utilization, largely driven by prescribing for respiratory tract infections, raised significant concerns regarding sustainability and the potential acceleration of antimicrobial resistance (Isler et al., 2019; Torumkuney et al., 2022).

In response, the Turkish Ministry of Health launched the National Action Plan for Rational Drug Use in 2014, placing rational antibiotic prescribing at the center of national health policy (Karaali, 2025; Gönen et al., 2021). Beginning in 2014, a series of coordinated antimicrobial stewardship interventions were implemented nationwide. A key regulatory component was the strict enforcement of prescription-only dispensing of systemic antibiotics, which became fully effective in 2016 and aimed to eliminate widespread self-medication practices (Global Health Security Index, 2021; Llor et al., 2024). In parallel, national clinical guidelines for common infectious conditions were disseminated to promote first-line, narrow-spectrum antibiotic therapy and to discourage unnecessary prescribing. The integration of mandatory ICD-10 diagnostic coding into the national electronic prescribing system enabled closer monitoring of prescribing indications and facilitated audit-and-feedback mechanisms targeting high-prescribing physicians (Özcebe et al., 2022).

These regulatory measures were supported by extensive educational initiatives directed at healthcare providers. Nationwide continuing medical education programs emphasized rational antibiotic use and adherence to evidence-based guidelines, an approach shown to significantly reduce inappropriate prescribing in outpatient settings (Zheng et al., 2022). Public awareness campaigns were also introduced to address patient expectations and demand for antibiotics, particularly for viral upper respiratory tract infections, which had historically driven high antibiotic use in Türkiye (World Health Organization, 2015; Azap et al., 2019). Together, regulatory enforcement, guideline dissemination, prescriber education, public engagement, and systematic prescription monitoring constituted the core components of Türkiye’s national antimicrobial stewardship strategy implemented after 2014.

Primary care was a central focus of these interventions, as family physicians are responsible for the majority of outpatient antibiotic prescriptions. In many healthcare systems, including Türkiye, approximately 80–90% of antibiotics are prescribed in the community, predominantly for respiratory tract infections (Llor and Bjerrum, 2014). Targeting prescribing practices in family medicine was therefore expected to have a substantial impact on national antibiotic consumption patterns and, by extension, on antimicrobial resistance.

Although the present analysis covers the pre–COVID-19 period (2011–2019), it is important to acknowledge that the COVID-19 pandemic has subsequently altered antibiotic prescribing patterns worldwide. Several studies have reported increased antibiotic use during the pandemic, often in the absence of confirmed bacterial infection, raising concerns about further acceleration of antimicrobial resistance (World Health Organization, 2004). These developments highlight the importance of understanding and sustaining effective stewardship policies that were established prior to the pandemic, such as those evaluated in this study.

This study evaluates temporal trends in antibiotic prescribing in Türkiye’s primary care system between 2011 and 2019, a period encompassing the implementation of major national stewardship interventions. Using nationwide prescription data, we assess changes in antibiotic prescribing rates, consumption levels, antibiotic class distribution benchmarked against the WHO AWaRe classification, and associated economic impacts. Although the study is descriptive and does not permit causal inference, documenting these national trends provides valuable insight into the potential effects of coordinated stewardship policies and offers lessons for future antimicrobial resistance containment efforts in Türkiye and comparable settings.

## Methods

### Study design and data source

We conducted a nationwide, descriptive, retrospective analysis of outpatient antibiotic prescribing in Türkiye’s primary care (family medicine) system covering the period from 2011 to 2019. The primary data source was the Reçete Bilgi Sistemi (RBS), the centralized Prescription Information System maintained by the Turkish Medicines and Medical Devices Agency (TİTCK). The RBS database captures all prescriptions issued by family physicians across all 81 provinces, as electronic prescribing has been universally implemented in primary care throughout the study period. As a result, the dataset provides comprehensive national coverage of outpatient antibiotic use in the primary care setting. Importantly, the RBS records only prescriptions issued through the electronic prescribing system; antibiotics dispensed without a medical prescription (over-the-counter sales, which were legal in Türkiye until 2016 and reportedly remained partially available thereafter) are not captured. The analysis therefore characterizes prescriber behavior and prescribed antibiotic exposure rather than total community antibiotic exposure, and post-2016 trends must be interpreted in this light (see Discussion).

### Data collection and variables

Aggregated annual data were obtained from the RBS through an official request to TİTCK and were supplemented with publicly available national reports. The analysis focused on several key domains of antibiotic prescribing and utilization (Table 1).

**Table 1:** Data elements obtained from the Prescription Information System (RBS) for analysis of antibiotic prescribing in Turkish primary care, 2011–2019.

Category	Variable/Indicator	Years available	Description
Prescription counts	Total prescriptions; prescriptions containing ≥1 antibiotic (ATC J01)	2011–2019	Annual totals; used to calculate antibiotic prescription rate.
Antibiotic prescription rate	% of all prescriptions including ≥1 antibiotic	2011–2019	Calculated as antibiotic prescriptions ÷ total prescriptions × 100.
Antibiotic consumption	DID (defined daily doses per 1,000 inhabitants per day) for ATC J01 overall	2011–2019	Based on WHO ATC/DDD methodology.
Antibiotic consumption by class	DID values for ATC level-3 (e.g., J01C penicillins, J01D cephalosporins, J01F macrolides, etc.) and level-5 (individual agents)	2011–2019	Provides stratified consumption patterns by class and substance.
Demographic subgroup data	Prescriptions and antibiotic prescriptions by sex (male/female) and by age groups (0–6, 7–19, 20–64, ≥65 years)	2013–2019	Allows calculation of subgroup-specific antibiotic prescription rates.
Diagnosis data	Top 10 ICD-10 codes associated with antibiotic prescriptions	2013–2019	Enables analysis of indications (e.g., respiratory infections) and changes over time.
Drug utilization & costs	Total outpatient drug volume (packages/boxes) and expenditures (TL); antibiotic share of both	2011–2019	Calculates annual proportion of antibiotics in overall prescribing volume and costs.
AWaRe classification	Distribution of antibiotic consumption into Access, Watch, Reserve categories (based on ATC-5 codes, WHO AWaRe list 2019)	2011–2019	Evaluates alignment with WHO stewardship benchmarks (≥60% Access).
Cost impact	Projected vs. actual antibiotic expenditure, comparing 2011 baseline to 2018/2019	2011–2019	Estimates financial savings from reduced antibiotic use.

First, prescription counts were extracted, including the total number of prescriptions issued annually and the number of prescriptions containing at least one systemic antibiotic classified under ATC code J01. Based on these data, the annual antibiotic prescription rate was calculated as the proportion of total prescriptions that included one or more antibiotics.

Second, antibiotic consumption was estimated using the World Health Organization (WHO) Anatomical Therapeutic Chemical/Defined Daily Dose (ATC/DDD) methodology and expressed as defined daily doses per 1,000 inhabitants per day (DID) (WHO Collaborating Centre for Drug Statistics Methodology, 2025). Consumption estimates were calculated for all systemic antibiotics combined (ATC J01) and further stratified by ATC level-3 classes, such as penicillins (J01C), cephalosporins (J01D), macrolides (J01F), fluoroquinolones (J01M), and other relevant groups, as well as by ATC level-5 individual agents where applicable. Antibiotic consumption was quantified using the WHO ATC/DDD methodology and expressed as defined daily doses per 1,000 inhabitants per day. For each calendar year, the total number of defined daily doses (DDDs) dispensed for systemic antibiotics (ATC J01) was calculated based on aggregated prescription data. DID values were then derived by dividing the annual total DDDs by the mid-year population and the number of days in the year, and multiplying by 1,000, in accordance with WHO recommendations. This standardized metric enables normalization of antibiotic consumption to population size and facilitates comparisons across years and antibiotic classes.

Third, demographic distributions of antibiotic prescribing were examined for the period 2013–2019, for which such data were available. Antibiotic prescriptions were analyzed by patient sex (male and female) and by age group (0–6 years, 7–19 years, 20–64 years, and ≥65 years). These data enabled the calculation of subgroup-specific antibiotic prescription rates and the assessment of temporal trends across demographic categories.

Fourth, diagnostic information linked to antibiotic prescriptions was evaluated. For each year between 2013 and 2019, the ten most frequent ICD-10 diagnostic codes associated with antibiotic prescribing were extracted. This allowed assessment of changes over time in the clinical indications for which antibiotics were prescribed, with particular attention to respiratory tract infections.

Fifth, overall outpatient drug utilization and expenditures were analyzed. Data on the total number of outpatient prescriptions for all medications, the number of antibiotic packages (boxes) dispensed, and corresponding expenditures in Turkish Lira (TL) were obtained for each study year. The annual share of antibiotics in total outpatient drug volume and costs was calculated to assess changes in the relative contribution of antibiotics to overall pharmaceutical use.

Sixth, antibiotics were categorized according to the WHO Access, Watch, and Reserve (AWaRe) classification using ATC level-5 codes and the 2019 WHO AWaRe list (World Health Organization, 2023). Access antibiotics include first-line agents with lower resistance potential, Watch antibiotics comprise broader-spectrum agents with higher resistance risk, and Reserve antibiotics represent last-resort options not typically used in primary care. The proportion of antibiotic consumption in each AWaRe category was estimated annually based on DDDs. Antibiotics were categorized according to the World Health Organization (WHO) Access, Watch, and Reserve (AWaRe) classification using ATC level-5 substance codes and the WHO AWaRe list. A single, standardized mapping table was applied uniformly across the entire dataset. No institution-specific or locally customized AWaRe categorizations were used in this analysis. This approach ensured that all reported Access, Watch, and Reserve proportions reflect the WHO-defined categories and remain directly comparable across time and settings, avoiding potential inconsistencies that may arise from local adaptations of stewardship classifications.

Finally, a cost impact analysis was performed to estimate the economic implications of changes in antibiotic use. The year 2011 was used as a baseline, and 2018 was selected as a late-intervention year, consistent with previous national analyses (Westerling et al., 2020). Projected antibiotic expenditures for 2018 were calculated under the hypothetical assumption that antibiotic utilization levels had remained at 2011 values. The difference between projected and actual expenditures was interpreted as cost savings attributable to reduced antibiotic use. A confirmatory analysis was conducted for 2019. All cost estimates are presented in nominal Turkish Lira without adjustment for inflation.

### Statistical analysis

Descriptive statistics were used to summarize annual trends in antibiotic prescribing and consumption. Outcomes were reported as counts, percentages, and DID values. Two outcomes were pre-specified as primary: the annual antibiotic prescription rate (% of prescriptions containing ≥1 antibiotic) and the total annual systemic antibiotic consumption (DID, ATC J01). All other indicators (ATC-3 class, AWaRe category, age- and sex-stratified rates, ICD-10 diagnoses, and cost) were treated as secondary, descriptive outcomes. To assess whether changes in the temporal trend coincided with the consolidation of national stewardship policies, segmented (interrupted time-series) regression following the framework of Wagner et al. (J Clin Pharm Ther 2002) and Bernal et al. (Int J Epidemiol 2017) was applied to the two primary outcomes. The intervention point was set at 2016, when prescription-only dispensing of systemic antibiotics was strictly enforced and mandatory ICD-10 coding within the e-prescribing system was fully consolidated, capping the regulatory bundle initiated by the 2014 National Action Plan. The model estimated the pre-intervention slope, the immediate level change at 2016 and the change in slope after 2016; the post-intervention slope was derived as their sum. Newey–West heteroskedasticity- and autocorrelation-consistent standard errors (lag = 1) were used to address residual autocorrelation given the small annual time series. A sensitivity analysis with the intervention point set at 2014 (launch of the National Action Plan) yielded directionally consistent results. Statistical analyses were performed in Python 3.11 with statsmodels v0.14; two-sided *p* values <0.05 were considered statistically significant. Key outcomes are presented in tables and figures, including annual antibiotic prescription rates, consumption trends by antibiotic class, and demographic subgroup analyses. Where appropriate, findings were contextualized using external benchmarks, such as the WHO target that at least 60% of national antibiotic consumption should fall within the Access category. All analyses were conducted using aggregated, de-identified data.

### Ethical considerations and analytical limitations

The study protocol was approved by the Ethics Committee of Bilkent City Hospital (Decision No: E1/902/2020). As the analysis was based exclusively on anonymized, aggregated national prescription data, individual informed consent was not required.

This study is observational and ecological in nature. Although segmented (interrupted time-series) regression has been applied to the two primary outcomes (Statistical Analysis), no concurrent comparator population was available; residual confounding by secular trends, public-awareness campaigns, or coding artefacts cannot be excluded, and causal attribution of observed changes to any individual stewardship component remains beyond the scope of this design. The findings should be interpreted as descriptive associations over time, which may reflect the combined influence of multiple concurrent factors in addition to national stewardship policies.

## Results

### National trends in antibiotic prescribing and consumption (2011–2019)

**Figure 1:**
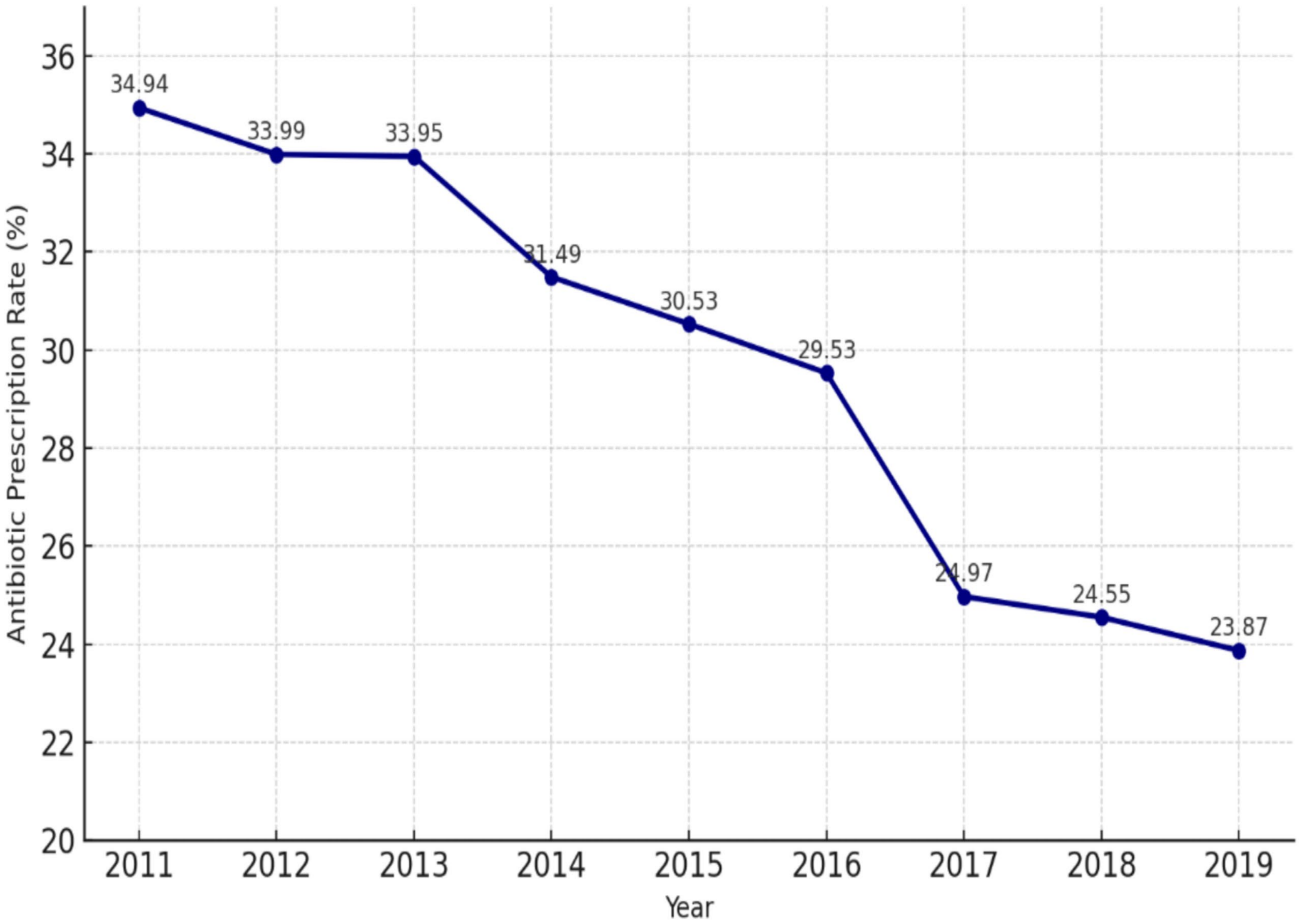
Trend in the percentage of primary care prescriptions containing an antibiotic in Türkiye, 2011–2019. The dotted line indicates the timing of key interventions (2014–2017 National Action Plan implementation).

**Figure 2:**
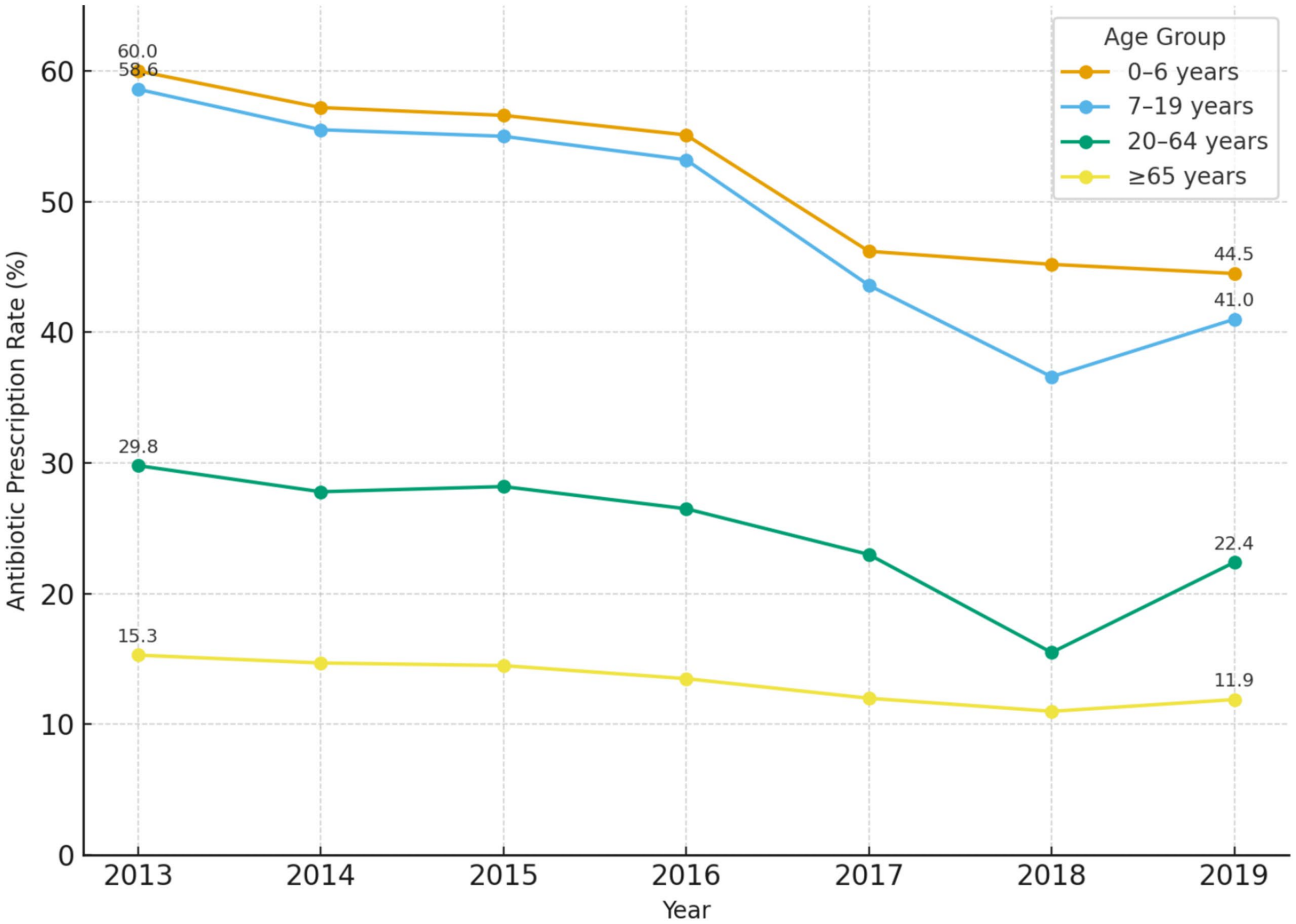
Antibiotic prescription rates were consistently highest in children aged 0–6 years, followed by adolescents, while the elderly had the lowest rates. All age groups demonstrated downward trends over time, with the most pronounced reductions observed after 2016.

Between 2011 and 2019, antibiotic prescribing in Türkiye’s primary care settings declined substantially. In 2011, 34.9% of all prescriptions issued by family physicians contained at least one antibiotic, whereas by 2019 this proportion had decreased to 23.9%, corresponding to an absolute reduction of approximately 11 percentage points. A similar downward trend was observed across all age groups (Figures 1, 2). The decline became more pronounced after 2016, when the antibiotic prescription rate fell to 29.5%, further decreasing to approximately 25.0% in 2017, and reaching its lowest level in 2019 (23.87%) (Figure 1).

**Figure 3:**
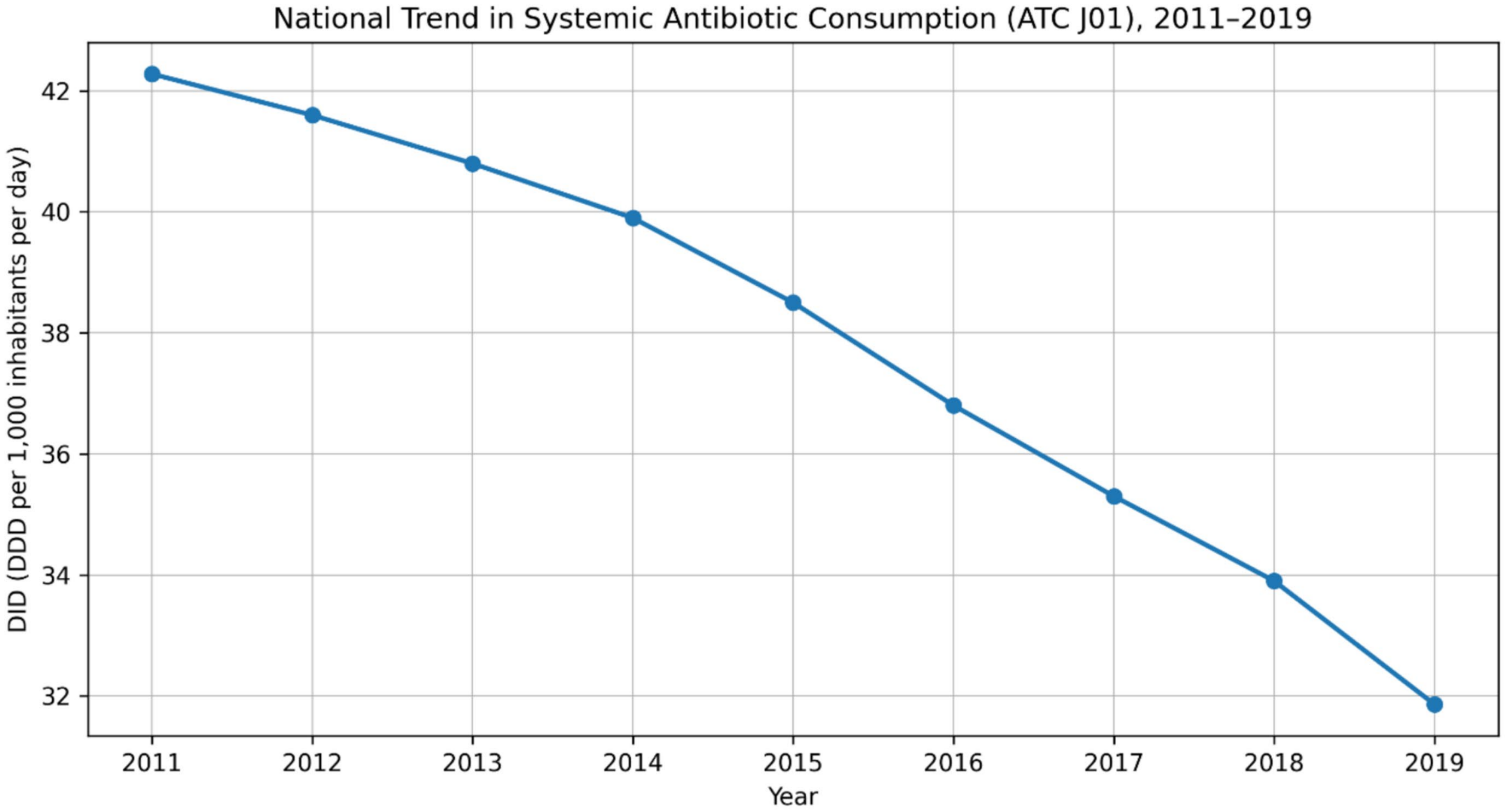
National trend in systemic antibiotic consumption (ATC J01) in Türkiye’s primary care, expressed as defined daily doses per 1,000 inhabitants per day (DID), 2011–2019.

Outpatient antibiotic consumption also declined over the study period. In 2011, total systemic antibiotic use in primary care was 42.28 DID (defined daily doses per 1,000 inhabitants per day). This metric remained within the 39–42 DID range through 2014, decreased to approximately 35.3 DID in 2017, and reached 31.86 DID in 2019 (Figure 3), corresponding to an approximate 25% reduction from 2011 to 2019.

Antibiotic consumption by ATC level-3 class was as follows. Penicillins (J01C, including penicillin/beta-lactamase inhibitor combinations) were 17.32 DID in 2011, reached 12.40 DID in 2018, and were 13.57 DID in 2019. Cephalosporins and other beta-lactams (J01D) were 14.11 DID in 2011 and 8.54 DID in 2019. Macrolides, lincosamides, and streptogramins (J01F) were 3.90 DID in 2011 and 4.02 DID in 2019, with a peak around 2015 (4.71 DID). Fluoroquinolones (J01M) were 3.61 DID in 2011 and 2.78 DID in 2019 (approximately 23% lower). Tetracyclines (J01A) were approximately 1.3–1.4 DID in 2011–2012 and 1.27 DID in 2019. Trimethoprim/sulfamethoxazole (J01E) declined from 0.46 DID in 2011 to 0.28 DID in 2019. Other antibiotics (J01X, including nitrofurantoin, fosfomycin, and metronidazole for systemic use) were 1.42 DID in 2011 and approximately 1.36 DID in 2019. Aminoglycosides (J01G) were near 0 DID throughout. Antibiotic combinations (J01R) were 0.13 DID in 2017 and essentially 0 by 2019. By the end of 2019, beta-lactams (penicillins and cephalosporins combined) constituted approximately 69% of total DID and macrolides approximately 13%.

### AWaRe category distribution (2011–2019)

Antibiotic use in Türkiye’s primary care was mapped to the WHO AWaRe framework. In 2019, Access group antibiotics accounted for approximately 50–55% of total outpatient antibiotic consumption by DID (depending on the classification of certain agents). Common Access antibiotics in primary care included amoxicillin, amoxicillin-clavulanate, first-generation cephalosporins (e.g., cephalexin), tetracyclines (doxycycline), trimethoprim-sulfamethoxazole, nitrofurantoin, and clindamycin. Watch group antibiotics accounted for nearly half of use and included second- and third-generation cephalosporins (cefuroxime, cefixime, cefdinir), macrolides (azithromycin, clarithromycin), and fluoroquinolones. Reserve group antibiotics (e.g., linezolid, carbapenems, colistin) were essentially not used in outpatient primary care. The WHO benchmark of ≥60% Access-category antibiotic use by 2023 is presented as an external reference point.

In addition to prescription-based indicators, defined daily doses per 1,000 inhabitants per day (DID) were used as the primary quantitative measure of population-level antibiotic consumption. DID trends provide a standardized assessment of antibiotic volume normalized to population size and complement prescription rates by capturing changes in overall consumption intensity over time. Accordingly, the observed reduction from 42.28 DID in 2011 to 31.86 DID in 2019 reflects a substantial quantitative decline in outpatient antibiotic use at the national level (Figure 3). DID is therefore the principal quantitative indicator of population-level antibiotic exposure in the present study; the antibiotic prescription rate is retained as a complementary indicator of prescribing behavior at the individual encounter level rather than as a parallel measure of the same construct, and the two metrics convey conceptually distinct dimensions of the prescribing pattern.

### Prescribing by sex (2013–2019)

From 2013 onward, prescribing patterns by sex were available. Annual totals for prescriptions and antibiotic prescriptions by sex, together with the percentage of prescriptions containing antibiotics, are provided in Table 2.

**Table 2:** Annual total prescriptions, antibiotic prescriptions, and percentage of prescriptions with antibiotics by patient sex in Turkish primary care (2013–2019).

Year	Total Rx (Male)	Total Rx (Female)	Antibiotic Rx (Male)	Antibiotic Rx (Female)	% Antibiotic Rx (Male)	% Antibiotic Rx (Female)
2013	47,426,669	69,539,367	17,516,244	22,190,109	36.93%	31.91%
2014	55,888,149	82,225,432	19,156,718	24,325,928	34.28%	29.58%
2015	53,210,296	76,774,173	17,916,622	22,526,943	33.67%	29.34%
2016	54,985,853	79,240,965	17,566,543	22,057,972	31.95%	27.84%
2017	58,784,056	82,825,449	15,560,144	19,798,493	26.47%	23.90%
2018	64,728,015	90,543,412	16,758,888	21,360,752	25.89%	23.59%
2019	70,358,826	97,771,189	17,588,939	22,546,082	25.00%	23.06%

### Prescribing by age group (2013–2019)

Age-group prescribing patterns were evaluated for 2013–2019. The annual proportion of prescriptions containing an antibiotic by age category is shown in Table 3.

**Table 3:** Proportion of prescriptions containing an antibiotic by patient age group in primary care, 2013–2019.

Year	0–6 years	7–19 years	20–64 years	≥65 years
2013	60.0%	58.6%	29.8%	15.3%
2014	57.2%	55.5%	27.8%	14.7%
2015	56.6%	55.0%	28.2%	14.5%
2016	55.1%	53.2%	26.5%	13.5%
2017	46.2%	43.6%	23.0%	12.0%
2018	45.2%	36.6%	15.5%	11.0%
2019	44.5%	41.0%	22.4%	11.9%

### Diagnosis patterns (ICD-10 coding linked to antibiotic prescriptions; 2013–2019)

ICD-10 codes associated with antibiotic prescriptions were evaluated using the top 10 codes annually (2013–2019). For acute upper respiratory infection, unspecified (J06.9), the share of antibiotic prescriptions was 8.54% in 2016, 4.62% in 2017, 4.31% in 2018, and 4.09% in 2019. For unspecified acute pharyngitis (J02.9), the share of antibiotic prescriptions was 7.92% in 2016 and 3.14% in 2017, and the code did not appear among the top 10 diagnoses for antibiotic prescriptions after 2017. Empirical antibiotic prescribing for acute pharyngitis was reported as occurring in approximately 8–9% of all antibiotic-containing prescriptions during 2013–2015.

### Overall prescription volume, antibiotic share, and cost impact

Total primary care prescription volume increased between 2013 and 2019, from approximately 117 million to 168 million prescriptions per year (approximately 44%). Over the same period, the number of antibiotic-containing prescriptions remained approximately 39–40 million per year.

Antibiotics represented 13.1% of all outpatient drug boxes in 2011 and 8.0% in 2019. Antibiotics accounted for 10.7% of total outpatient drug spending in 2011 and 4.7% in 2019. The absolute number of antibiotic packages (boxes) dispensed annually declined from approximately 218 million in 2011 to 167 million in 2019.

For cost impact, projected outpatient antibiotic expenditure for 2018 under 2011 utilization levels was approximately 3.0 billion TL, whereas actual antibiotic spending in 2018 was approximately 1.29 billion TL. The difference between projected and actual spending was approximately 1.7 billion TL. A confirmatory analysis was conducted for 2019.

### Segmented (interrupted time-series) regression of the two primary outcomes

**Figure 4:**
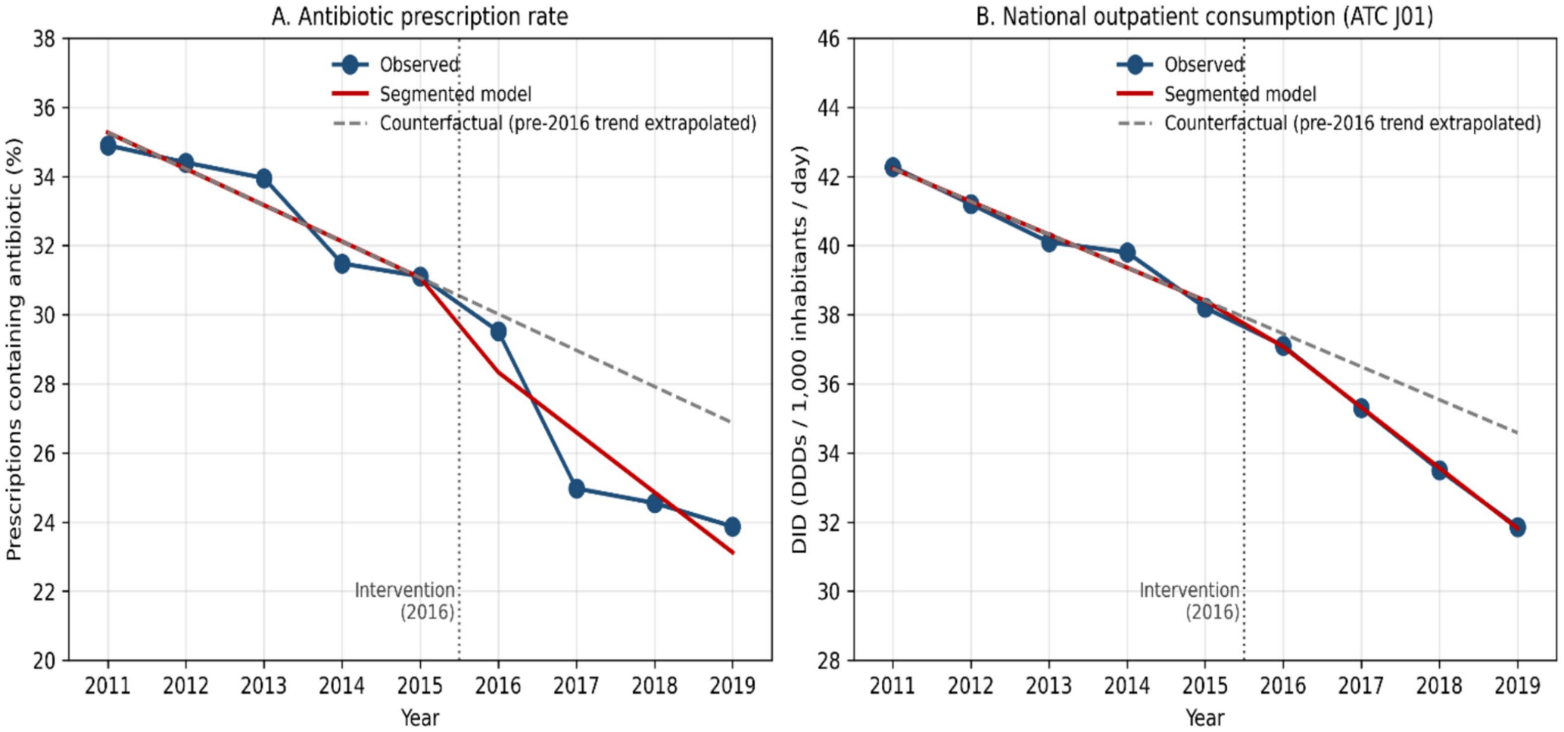
Segmented (interrupted time-series) regression of **(A)** the annual antibiotic prescription rate and **(B)** total national outpatient antibiotic consumption (ATC J01, DID) in Türkiye, 2011–2019. Solid line: fitted segmented model; dashed line: counterfactual obtained by extrapolating the pre-2016 trend; vertical dotted line: intervention point (2016, full enforcement of prescription-only dispensing of systemic antibiotics and consolidation of mandatory ICD-10 coding in the e-prescribing system).

Segmented regression with the intervention point set at 2016 demonstrated that the rate of decline in antibiotic use accelerated significantly after the consolidation of national stewardship measures (Figure 4). For the antibiotic prescription rate, the pre-intervention (2011–2015) slope was −1.05 percentage points per year (95% CI − 1.23 to −0.88; *p* < 0.001); the slope changed by an additional −0.69 percentage points per year after 2016 (95% CI − 1.44 to +0.06; *p* = 0.072), yielding a post-intervention slope of −1.74 percentage points per year (approximately 1.7-fold steeper than the pre-intervention trajectory). For total systemic antibiotic consumption, the pre-intervention slope was −0.96 DID per year (95% CI − 1.04 to −0.87; *p* < 0.001); the slope changed by −0.80 DID per year after 2016 (95% CI − 0.88 to −0.72; *p* < 0.001), giving a post-intervention slope of −1.75 DID per year. A small positive immediate level change of +0.42 DID was observed at 2016 (95% CI + 0.11 to +0.72; *p* = 0.007), which is consistent with the migration of formerly over-the-counter purchases into the recorded prescription system once over-the-counter dispensing was prohibited (see Discussion). Model fit was high for both outcomes (R^2^ = 0.96 and 0.997, respectively). Annual mid-year population values used to derive DID are provided in Supplementary Table S1.

## Discussion

In this nationwide analysis of primary care antibiotic prescribing in Türkiye, antibiotic use declined substantially between 2011 and 2019. The proportion of family practice prescriptions containing an antibiotic fell from approximately 35 to 24%, and community consumption decreased by roughly one quarter. These changes occurred during a period in which the Ministry of Health implemented comprehensive stewardship measures, consistent with evidence that coordinated national actions; regulation, prescriber and public education, guideline dissemination, and surveillance with feedback, can reduce unnecessary use.

Several aspects of Türkiye’s experience warrant attention. The acceleration in decline after 2014 is temporally consistent with the National Action Plan for Rational Drug Use (2014–2017) and subsequent activities, suggesting a policy-concordant change in the series (Türkiye İlaç ve Tıbbi Cihaz Kurumu, Akılcı İlaç Kullanımı Dairesi, 2014). Prior to these reforms, Türkiye ranked among the highest antibiotic consumers in the WHO European Region, with total use near 42.3 DID and co-amoxiclav accounting for approximately 30.7% of all antibiotics (T.C. Sağlık Bakanlığı, Türkiye İlaç ve Tıbbi Cihaz Kurumu, 2011; Versporten et al., 2014). The marked reduction between 2016 and 2017 aligns with prohibition and strengthened enforcement of over-the-counter sales of systemic antibiotics, an intervention intended to curb access and self-medication (Global Health Security Index, 2021; Özcebe et al., 2022). This temporal pattern parallels findings from a systematic review reporting that national measures such as oversight committees, prescribing restrictions, and updated guidelines are associated with reductions in antibiotic use (Lim et al., 2020). Formal segmented regression on the present series corroborated this temporal pattern: both primary outcomes showed a significant change in slope after 2016 relative to the pre-existing trajectory, with the post-intervention rate of decline approximately 1.7-fold steeper than the pre-intervention slope (Figure 4, Results). The small positive immediate level change in DID at 2016 (+0.42 DID) is unlikely to represent a true rise in exposure and most plausibly reflects the absorption of formerly over-the-counter purchases into the prescription record once OTC sales were prohibited—a pattern that, if anything, would tend to underestimate rather than overstate the true population-level reduction in antibiotic exposure.

Interpretation remains constrained by the observational, single-country nature of the study, and the contribution of any individual policy component cannot be isolated in the absence of a concurrent comparator population, even after segmented regression. To support plausibility, Mexico’s 2010 transition to prescription-only antibiotics was followed by an approximately 29% decline in outpatient consumption in time-series studies, providing an external analogue for Türkiye’s 2016 intervention (Santa-Ana-Tellez et al., 2013). Conversely, qualitative work from Türkiye during and after the ban has described residual non-prescription access and persistent patient demand, indicating that enforcement requires reinforcement through provider training and public campaigns to sustain reductions (Özcebe et al., 2022). Taken together, the timing and direction of change are concordant with policy implementation and international experience, while more robust study designs would be needed to quantify effect sizes attributable to specific components.

Education targeting clinicians and the public represented a central element of the national approach, and the downward trajectory observed here coincides with broader evidence that educational interventions can improve prescribing. Reviews have shown that education, particularly when combined with other stewardship elements, can reduce prescribing and improve guideline adherence; Roque et al. reported reductions of up to 41% across included studies, alongside improved adherence (Roque et al., 2014). Baseline public awareness in Türkiye was reported to be limited: a 2014 Ankara survey found that 64.3% reported self-medicating with antibiotics, 64% would request antibiotics for flu-like illness, 64.9% kept antibiotics at home, and 87% could purchase antibiotics without a prescription (Gül et al., 2014). Public-facing national messaging was therefore implemented to reduce inappropriate demand and support guideline-concordant prescribing (Gül et al., 2014). Concurrently, the shift away from nonspecific diagnostic labels, including fewer unspecified URIs when antibiotics were prescribed, is consistent with stewardship approaches emphasizing tracking, reporting, and decision support. Quality-improvement projects coupling education with coding feedback have reported reductions in URI-associated prescribing in primary care (Centers for Disease Control and Prevention, 2024; Sathe et al., 2024). At the same time, education alone yields variable effects unless embedded within broader system-level measures such as audit/feedback, access restriction, and point-of-care testing (Rocha et al., 2022). Within these constraints, the observed timing and direction of change are consistent with a multifaceted strategy rather than a single isolated intervention.

Despite progress, Türkiye began from a high baseline and remained a relatively high antibiotic user compared with international standards. In 2019, outpatient consumption was approximately 31.9 DID, exceeding the 10–25 DID range reported in many EU countries (European Centre for Disease Prevention and Control, 2020). WHO and ECDC reports have placed Türkiye among the top consumers in the region, although recent data suggest that the gap has narrowed. These findings indicate that reductions achieved during the study period occurred within a context of persistently elevated consumption relative to many comparator countries. For 2019 specifically, ECDC ESAC-Net data show community antibiotic consumption (ATC J01, expressed as DID) of approximately 9.3 in the Netherlands, 10.3 in Sweden, 11.6 in Germany, 17.1 in the United Kingdom, 17.7 in Italy, 23.3 in France, 25.6 in Romania and 32.0 in Greece (European Centre for Disease Prevention and Control, 2020). Türkiye’s 2019 value of 31.9 DID therefore remained at the upper end of the WHO European Region—comparable to Greece and approximately three-fold higher than the level achieved in the Northern European countries with the most established stewardship programmes—underscoring that meaningful improvement has been achieved within a context of persistently elevated consumption.

Beyond overall volume, antibiotic spectrum distribution is central to stewardship goals. In our dataset, Watch antibiotics accounted for roughly half of outpatient use, consistent with WHO concerns that oral Watch agents remain overused and should be displaced by Access drugs where clinically appropriate; cross-country analyses of pediatric oral antibiotic sales have similarly shown disproportionate Watch-group consumption in middle- and high-income settings (Hsia et al., 2019; Abdelsalam Elshenawy et al., 2023; Zanichelli et al., 2023). Substitution is possible in common outpatient scenarios: for respiratory infections requiring antibiotics, guidelines often endorse narrow-spectrum amoxicillin as first line for selected presentations, while amoxicillin–clavulanate, although categorized as Access by WHO due to its essential role, is broader in spectrum and may have greater ecological impact (Zanichelli et al., 2023; National Institute for Health and Care Excellence (NICE), 2019; National Institute for Health and Care Excellence (NICE), 2018). Similarly, for uncomplicated urinary tract infection, nitrofurantoin (Access) is recommended first line, whereas fluoroquinolones (Watch) are generally discouraged (National Institute for Health and Care Excellence (NICE), 2018). Guideline nuances must be acknowledged. For example, for acute bacterial rhinosinusitis in adults, IDSA guidelines recommend amoxicillin–clavulanate rather than amoxicillin monotherapy in many settings due to *β*-lactamase–producing pathogens (Chow et al., 2012; World Health Organization, 2025). WHO also emphasizes that AWaRe categories reflect stewardship and procurement priorities, and that classification of agents such as amoxicillin–clavulanate can be context dependent (World Health Organization, 2023; Abdelsalam Elshenawy et al., 2023; World Health Organization, 2025). In this framework, the observed declines in quinolone use and modest shifts in prescribing patterns are compatible with stewardship objectives, while the WHO target of at least 60% Access antibiotics remains an external benchmark motivating further efforts (Zanichelli et al., 2023; Tucker et al., 2024).

Age-group analyses provide additional context. The largest reductions occurred among children and adolescents, groups historically exposed to higher outpatient antibiotic prescribing and previously identified as a priority population for dedicated antimicrobial stewardship efforts in Türkiye (Khan and Karataş, 2021). In the 0–6 year age group, prescription rates fell from about 60 to 44% of visits. Pediatric stewardship programs in other settings have reported reductions in antibiotic consumption, healthcare costs, and resistant organism colonization (Pagano et al., 2023). Potential long-term benefits have been discussed in relation to early-life antibiotic exposure, including changes in gut microbiome diversity and increases in resistance gene abundance lasting months to years (Wurm et al., 2024). Evidence is not uniform, and a recent study in otherwise healthy children reported minimal microbiome perturbation after a single amoxicillin course at one month, suggesting variability by drug class and context (Benitez et al., 2025). These considerations support continued emphasis on guideline-concordant prescribing and targeted interventions in pediatric care.

By contrast, the decline among older adults (≥65 years) was smaller. In many systems, older adults receive more antibiotics due to higher infection prevalence or prophylaxis for chronic conditions (Olesen et al., 2018). Türkiye’s comparatively lower outpatient rates in this age group may reflect a greater share of visits for chronic disease management in primary care or shifting of more severe infections to hospital care. Polypharmacy may also influence prescribing decisions: nationwide data from Türkiye report that geriatric patients average 6.4 prescriptions annually and that 14.3% meet criteria for chronic polypharmacy, factors that can affect clinicians’ tolerance for adding new agents (Aydos et al., 2020). Additional studies are needed to determine whether observed patterns in older adults reflect appropriate restraint, different care pathways, or unmet need.

Diagnosis-linked coding patterns offer additional signals regarding prescribing context. The decline after 2016 in antibiotic use for “unspecified URI” and “unspecified pharyngitis” is consistent with recommendations discouraging antibiotics for colds and promoting clinical criteria and testing for bacterial pharyngitis. Such changes align with audit-and-feedback (A&F) approaches, which can alter prescribing when monitoring and benchmarking are implemented. A recent meta-analysis reported that primary-care A&F interventions reduced antibiotic volume by 11%, unnecessary initiation by 23%, and broad-spectrum use by 17% (Xu et al., 2025). However, effectiveness varies by design and implementation. A Swiss randomized trial of quarterly peer-benchmarking found no overall reduction in prescribing, with only a modest decline in quinolone prescribing, underscoring the need for carefully structured interventions (Schwartz et al., 2021). In the present study, the use of aggregated data precluded patient-level assessment of clinical appropriateness, but diagnosis-linked trends provide descriptive evidence on shifts in documentation patterns over time.

From a global perspective, Türkiye’s experience contributes to the evidence base indicating that national policy interventions can reduce community antibiotic consumption. Long-term stewardship programs in Sweden and the UK have achieved sustained declines in community prescribing through guideline dissemination, surveillance, and public engagement (Mölstad et al., 2008; UK Health Security Agency, 2024). South Korea’s national action plan in the mid-2010s was associated with reductions in Watch-group use in hospital and outpatient settings (Chae et al., 2022). These comparisons support the concept that integrated strategies combining regulation, education, surveillance, and accountability are more likely to yield sustained changes than partial measures (Huttner et al., 2013; World Health Organization, 2015).

It is important to acknowledge key limitations. First, the study is observational and descriptive. We did not apply an interrupted time series design or include a control group (such as a comparison country or unaffected condition); therefore, causality between interventions and outcomes cannot be established. While the timing and magnitude of changes are consistent with policy implementation, other contemporaneous factors may have influenced prescribing trends, including increasing global awareness of AMR, economic conditions, and changes in healthcare utilization patterns. An ITS analysis would be an appropriate next step to quantify policy-associated changes while accounting for baseline trends and autocorrelation.

Second, the analysis is restricted to primary care (family medicine) prescriptions and does not capture hospital prescribing or specialist outpatient practice. Accordingly, the results describe community prescribing trends rather than total national antibiotic use across all sectors. Potential sectoral shifts could not be assessed. Nonetheless, given that outpatient settings account for the majority of antibiotic use in many systems, the primary-care focus remains central to stewardship evaluation.

Third, patient-level clinical detail was unavailable, and appropriateness could not be directly assessed beyond diagnosis coding and aggregate prescribing patterns. The remaining antibiotic prescription rate in 2019 may still include inappropriate prescribing (e.g., antibiotics for bronchitis or viral URIs). Patient outcomes were not evaluated, and the analysis cannot address potential consequences such as complications of infections. Existing evidence from other settings has shown that reducing inappropriate antibiotics can be achieved without adverse outcomes, while decreasing adverse events.

A further methodological consideration concerns the indicators themselves. DID, although widely used and standardized across the WHO European Region, captures the volume of antibiotic exposure but not the spectrum or appropriateness of the agents prescribed; trends in DID can therefore remain stable, or even improve, while the AWaRe distribution worsens. We mitigated this limitation by reporting AWaRe-stratified consumption alongside total DID, but no single metric is sufficient on its own. Similarly, the antibiotic prescription rate is sensitive to changes in the denominator (overall prescribing volume), which expanded by approximately 44% between 2013 and 2019 in our dataset; part of the apparent decline in rate therefore reflects denominator dilution rather than absolute reduction in antibiotic-containing prescriptions, which remained relatively stable at 39–40 million per year. We have explicitly presented absolute counts alongside rates and DID for this reason.

Another limitation is that the analysis does not link prescribing reductions to antimicrobial resistance outcomes. The ideal evaluation would connect utilization trends with community resistance patterns of common pathogens and assess temporal relationships. This was outside the scope of the present dataset. Future studies integrating primary care prescribing data with microbiological surveillance, including phenotypic resistance profiles and, where available, molecular surveillance of antimicrobial resistance genes (ARGs), could provide a more direct assessment of the relationship between changes in antibiotic consumption and resistance dynamics.

We also note specific data constraints. Sex and age breakdowns were unavailable for 2011–2012, and those analyses therefore begin in 2013. Some anomalies were observed in age-specific trends, such as a temporary sharp decline in the adult group in 2018, which could reflect coding or data issues rather than a true sudden change; these patterns should be interpreted cautiously. Despite these constraints, the study leverages a comprehensive national dataset covering all primary care prescriptions over nearly a decade, minimizing selection bias and enabling stratification by demographic subgroups and antibiotic classes.

These findings have implications for clinicians and policymakers. For clinical practice, the observed trends underscore the relevance of guideline-concordant management of common outpatient infections and continued prioritization of Access-group antibiotics when clinically appropriate. For policymakers, Türkiye’s experience provides an example of a coordinated national strategy combining prescription-only regulations, e-prescribing with mandatory diagnostic coding, guideline dissemination, education, and feedback mechanisms. Continued monitoring via RBS and periodic feedback to prescribers can support sustained stewardship. Updating and extending national action plans remains important, and future work may consider extending stewardship efforts to additional sectors, including dentistry and hospital care, where antibiotic use also contributes to resistance selection.

While stewardship remains central to reducing unnecessary antibiotic exposure, antimicrobial resistance mitigation also depends on broader innovation and implementation strategies. In this context, future work may evaluate how stewardship efforts interface with emerging therapeutic and preventive approaches, including improved delivery strategies and novel antibacterial targets, while maintaining the primary focus on evidence-based interventions that reduce inappropriate prescribing at the population level.

## Conclusion

This nationwide analysis demonstrates that Türkiye achieved a substantial reduction in primary care antibiotic prescribing and consumption between 2011 and 2019. The proportion of prescriptions containing antibiotics declined markedly, and overall outpatient antibiotic use decreased by approximately one quarter, despite a concurrent increase in overall primary care prescribing volume. These changes coincided temporally with the implementation of coordinated national stewardship policies, including regulatory enforcement, restriction of over-the-counter antibiotic sales, integration of electronic prescribing with mandatory diagnostic coding, clinician and public education initiatives, and systematic monitoring with feedback. Segmented regression confirmed that the rate of decline accelerated significantly after 2016, although the descriptive design and absence of a contemporaneous comparator preclude attribution of these changes to any single intervention component.

Although meaningful progress was observed, several challenges remain. Türkiye continues to rank among the higher antibiotic-consuming countries in the WHO European Region, and Watch-group antibiotics still constitute a substantial share of outpatient use. Pediatric prescribing, while reduced, remains relatively high compared with other age groups. Differences in prescribing patterns among older adults highlight the need for further investigation into care pathways and appropriateness across age strata.

From a public health perspective, these findings underscore the importance of sustained, system-wide stewardship efforts to consolidate gains achieved during the study period. Continued prioritization of Access-group antibiotics when clinically appropriate, reinforcement of evidence-based guidelines, and ongoing surveillance of prescribing trends remain essential. Future research employing robust analytical designs and integrating prescribing data with antimicrobial resistance surveillance may further clarify the long-term impact of national stewardship initiatives on resistance patterns and patient outcomes.

## Data Availability

The raw data supporting the conclusions of this article will be made available by the authors, without undue reservation.
