# Supplementary material for: Trends in primary care antibiotic prescribing during the implementation of national stewardship policies: Türkiye, 2011–2019
Source: Front Microbiol. 2026 May 22;17:1762561. doi: 10.3389/fmicb.2026.1762561 (PMC13236919; doi:10.3389/fmicb.2026.1762561)
Supplement: Supplementary file 1 [file Table_1.DOCX]

**Supplementary Table S1.** Annual mid-year population, total systemic antibiotic consumption (DID; ATC J01) and antibiotic prescription rate used in the analysis, Türkiye 2011–2019.

| **Year** | **Mid-year population** **(TurkStat ADNKS)** | **DID** **(ATC J01)** | **Antibiotic prescription** **rate (%)** |
| --- | --- | --- | --- |
| 2011 | 74,724,269 | 42.28 | 34.90 |
| 2012 | 75,627,384 | 41.20 | 34.40 |
| 2013 | 76,667,864 | 40.10 | 33.95 |
| 2014 | 77,695,904 | 39.80 | 31.48 |
| 2015 | 78,741,053 | 38.20 | 31.11 |
| 2016 | 79,814,871 | 37.10 | 29.52 |
| 2017 | 80,810,525 | 35.30 | 24.97 |
| 2018 | 82,003,882 | 33.50 | 24.55 |
| 2019 | 83,154,997 | 31.86 | 23.87 |

*ADNKS: Address-Based Population Registration System; DID: defined daily doses per 1,000 inhabitants per day; ATC: Anatomical Therapeutic Chemical classification. Mid-year population values were obtained from the Turkish Statistical Institute (TurkStat) ADNKS public series (https://www.tuik.gov.tr/). DID was calculated as: (total annual J01 DDDs prescribed and dispensed via the national e-prescription system) / (mid-year population × 365) × 1,000. The antibiotic prescription rate is defined as the percentage of all primary-care prescriptions in the Reçete Bilgi Sistemi (RBS) containing at least one antibacterial agent for systemic use (ATC J01).*
